# 3-Benzyl­isochroman-1-one

**DOI:** 10.1107/S1600536809004164

**Published:** 2009-02-11

**Authors:** Tariq Mahmood Babar, Ghulam Qadeer, Nasim Hasan Rama, Muhammad Khawar Rauf, Wai-Yeung Wong

**Affiliations:** a Department of Chemistry, Quaid-i-azam University, Islamabad 45320, Pakistan; b Department of Chemistry, Hong Kong Baptist University, Waterloo Road, Kowloon Tong, Hong Kong, People’s Republic of China

## Abstract

In the mol­ecule of the title compound, C_16_H_14_O_2_, the aromatic rings are oriented at a dihedral angle of 78.49 (3)°. The heterocyclic ring adopts a twist conformation. In the crystal structure, inter­molecular C—H⋯O hydrogen bonds link the mol­ecules into chains along the *c* axis.

## Related literature

For related structures, see: Schmalle *et al.* (1982 ▶); Schnebel *et al.* (2003 ▶). For a description of the Cambridge Structural Database, see: Allen (2002 ▶). For bond-length data, see: Allen *et al.* (1987 ▶). For puckering parameters, see: Cremer & Pople (1975 ▶).
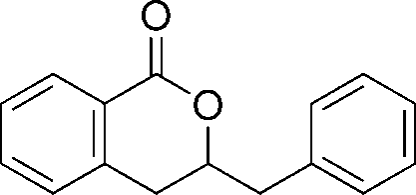


## Experimental

### 

#### Crystal data

C_16_H_14_O_2_
*M* *_r_* = 238.27
Monoclinic, 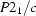

*a* = 12.503 (5) Å
*b* = 8.0200 (9) Å
*c* = 12.892 (5) Å
β = 102.43 (2)°
*V* = 1262.4 (7) Å^3^
*Z* = 4
Mo *K*α radiation
μ = 0.08 mm^−1^
*T* = 294 (2) K
0.32 × 0.26 × 0.21 mm

#### Data collection

Bruker SMART CCD area-detector diffractometer
Absorption correction: multi-scan (*SADABS*; Bruker, 2001 ▶) *T*_min_ = 0.820, *T*_max_ = 0.983
7148 measured reflections
3024 independent reflections
2546 reflections with *I* > 2σ(*I*)
*R*_int_ = 0.021

#### Refinement

*R*[*F* ^2^ > 2σ(*F* ^2^)] = 0.042
*wR*(*F* ^2^) = 0.136
*S* = 1.03
3024 reflections
164 parameters
H-atom parameters constrained
Δρ_max_ = 0.18 e Å^−3^
Δρ_min_ = −0.14 e Å^−3^

### 

Data collection: *SMART* (Bruker, 2001 ▶); cell refinement: *SAINT* (Bruker, 2002 ▶); data reduction: *SAINT*; program(s) used to solve structure: *SHELXS97* (Sheldrick, 2008 ▶); program(s) used to refine structure: *SHELXL97* (Sheldrick, 2008 ▶); molecular graphics: *ORTEP-3 for Windows* (Farrugia, 1997 ▶); software used to prepare material for publication: *SHELXTL* (Sheldrick, 2008 ▶).

## Supplementary Material

Crystal structure: contains datablocks I, global. DOI: 10.1107/S1600536809004164/hk2615sup1.cif

Structure factors: contains datablocks I. DOI: 10.1107/S1600536809004164/hk2615Isup2.hkl

Additional supplementary materials:  crystallographic information; 3D view; checkCIF report

## Figures and Tables

**Table 1:** Hydrogen-bond geometry (Å, °)

*D*—H⋯*A*	*D*—H	H⋯*A*	*D*⋯*A*	*D*—H⋯*A*
C9—H9*B*⋯O1^i^	0.97	2.53	3.2922 (18)	135

Symmetry code: (i) 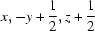.

**Fig. 1.**
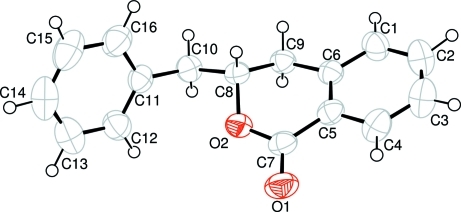
The molecular structure of the title molecule, with the atom-numbering scheme. Displacement ellipsoids are drawn at the 50% probability level.

**Fig. 2.**
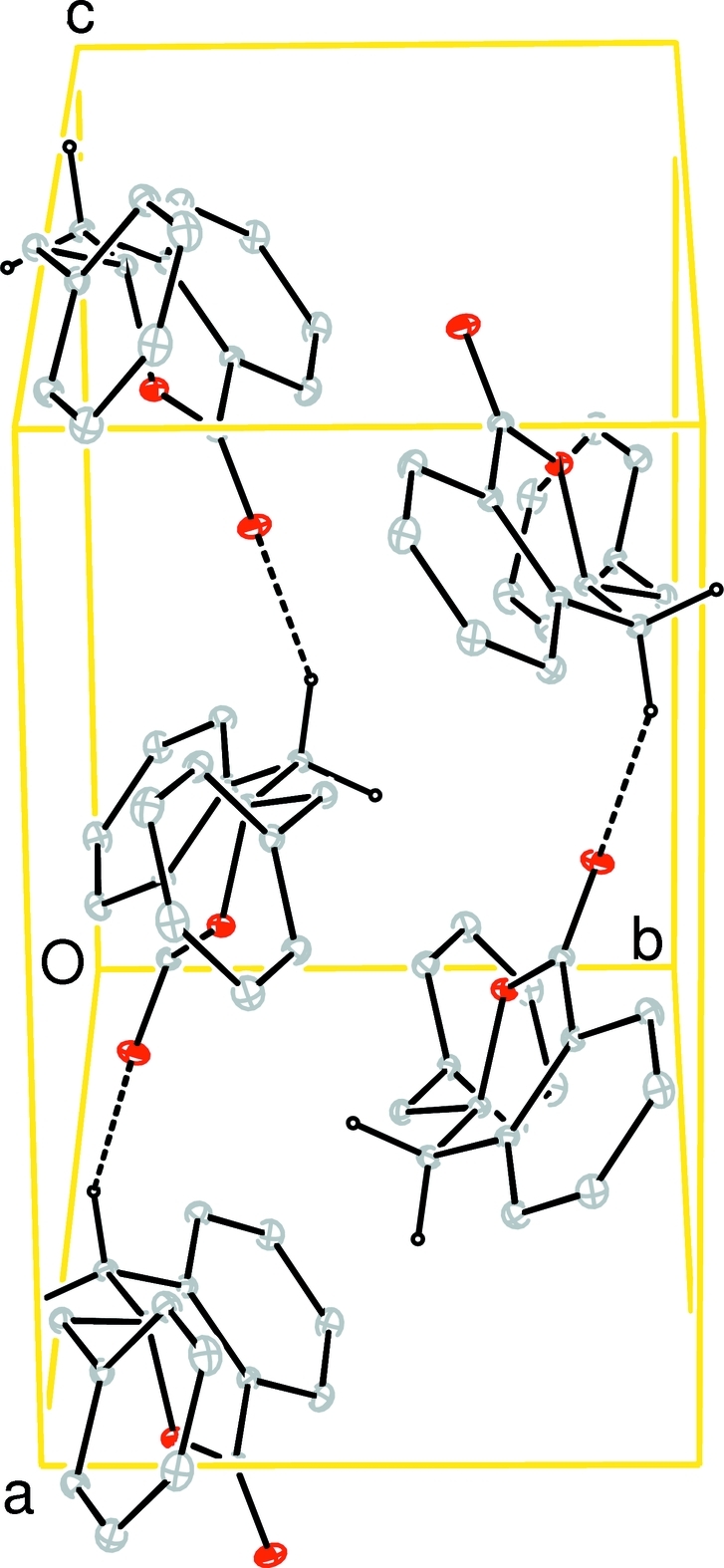
A partial packing diagram of the title compound. Hydrogen bonds are shown as dashed lines.

**Fig. 3.**
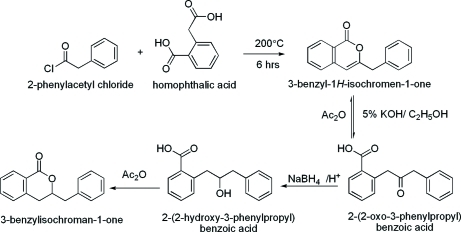
The formation of the title compound.

## supplementary crystallographic information

### Comment

The title compound was prepared in order to evalute its potential as antibacterial and antifungal agents. The CCDC search (Allen, 2002) showed that the crystal structures of rac-*exo*-tricarbonyl-(h6-3-phenyl isochromanone) -chromium (Schnebel *et al.*, 2003) and 3,4-dihydro-8-hydroxy-3-(4-hydroxy- phenyl)-isocoumarin (Schmalle *et al.*, 1982) have been reported, which have close resemblance as far as isochromane and attached phenyl ring is considered. We report herein the synthesis and crystal structure of the title compound.

In the molecule of the title compound (Fig. 1), the bond lengths (Allen *et al.*, 1987) and angles are within normal ranges. Rings A (C1-C6) and C (C11-C16) are, of course, planar, and they are oriented at a dihedral angle of 78.49 (3)°. Ring B (O2/C5-C9) is not planar, having total puckering amplitude, Q_T_, of 2.420 (3) Å and twisted conformation [φ = 151.98 (3)° and θ = 88.50 (3)°] (Cremer & Pople, 1975).

In the crystal structure, intermolecular C-H···O hydrogen bonds (Table 1) link the molecules into chains along the c axis, in which they may be effective in the stabilization of the structure.

### Experimental

As shown in Scheme 2, a mixture of homophthalic acid (1.98 g, 11.0 mmol) and 2-phenylacetyl chloride (7.08 g, 46 mmol) was heated under reflux for 6 h at 473 K. After concentration, the residue was chromatographed on silica gel column using petroleum ether (333–353 K) to give 3-benzyl-1*H*-isochromen-1-one. 2-(2-oxo-3-phenylpropyl) benzoic acid was obtained by refluxing a solution of 3-benzyl-1*H*-isochromen-1-one (4 g, 15.9 mmol) in ethanol (200 ml) and potassium hydroxide (5%,200 ml) for 6 h. NaBH4 (1.6 g) was added to a solution of 2-(2-oxo-3-phenylpropyl) benzoic acid (4.81 g, 17.8 mmol) in sodium hydroxide (1%, 180 ml) and the resulting solution was stirred overnight at room temperature. After being acidified with HCl, the whole mixture was extracted with dichloromethane (2 \ times 15 ml). Usual work-up gave crude racemic hydroxy-acid, 2-(2-hydroxy-3-phenylpropyl)benzoic acid, which was dissolved in acetic anhydride (5 ml) and heated under reflux for 2 h to get the title compound (yield; 73%, m.p. 605-606 K). The crude compound was purified by column chromatography on silica gel with petroleum ether and recrystallized in ethanol.

### Refinement

H atoms were positioned geometrically, with C-H = 0.93, 0.98 and 0.97 Å for aromatic, methine and methylene H, respectively, and constrained to ride on their parent atoms, with U_iso_(H) = 1.2U_eq_(C).

### Crystal data

**Table:**

C_16_H_14_O_2_	*F*(000) = 504
*M**_r_* = 238.27	*D*_x_ = 1.254 Mg m^−^^3^
Monoclinic, *P*2_1_/*c*	Melting point: 332(1) K
Hall symbol: -P 2ybc	Mo *K*α radiation, λ = 0.71073 Å
*a* = 12.503 (5) Å	Cell parameters from 1316 reflections
*b* = 8.0200 (9) Å	θ = 5.3–25.2°
*c* = 12.892 (5) Å	µ = 0.08 mm^−^^1^
β = 102.43 (2)°	*T* = 294 K
*V* = 1262.4 (7) Å^3^	Block, colorless
*Z* = 4	0.32 × 0.26 × 0.21 mm

### Data collection

**Table:**

Bruker SMART CCD area-detector diffractometer	3024 independent reflections
Radiation source: fine-focus sealed tube	2546 reflections with *I* > 2σ(*I*)
graphite	*R*_int_ = 0.021
ω and φ scans	θ_max_ = 28.5°, θ_min_ = 3.0°
Absorption correction: multi-scan (*SADABS*; Bruker, 2001)	*h* = −16→16
*T*_min_ = 0.820, *T*_max_ = 0.983	*k* = −10→10
7148 measured reflections	*l* = −10→17

### Refinement

**Table:**

Refinement on *F*^2^	Secondary atom site location: difference Fourier map
Least-squares matrix: full	Hydrogen site location: inferred from neighbouring sites
*R*[*F*^2^ > 2σ(*F*^2^)] = 0.042	H-atom parameters constrained
*wR*(*F*^2^) = 0.136	*w* = 1/[σ^2^(*F*_o_^2^) + (0.0818*P*)^2^ + 0.1131*P*] where *P* = (*F*_o_^2^ + 2*F*_c_^2^)/3
*S* = 1.02	(Δ/σ)_max_ < 0.001
3024 reflections	Δρ_max_ = 0.18 e Å^−^^3^
164 parameters	Δρ_min_ = −0.14 e Å^−^^3^
0 restraints	Extinction correction: *SHELXL97* (Sheldrick, 2008), Fc^*^=kFc[1+0.001xFc^2^λ^3^/sin(2θ)]^-1/4^
Primary atom site location: structure-invariant direct methods	Extinction coefficient: 0.058 (7)

### Special details

**Table:**

Geometry. All e.s.d.'s (except the e.s.d. in the dihedral angle between two l.s. planes) are estimated using the full covariance matrix. The cell e.s.d.'s are taken into account individually in the estimation of e.s.d.'s in distances, angles and torsion angles; correlations between e.s.d.'s in cell parameters are only used when they are defined by crystal symmetry. An approximate (isotropic) treatment of cell e.s.d.'s is used for estimating e.s.d.'s involving l.s. planes.
Refinement. Refinement of *F*^2^ against ALL reflections. The weighted *R*-factor *wR* and goodness of fit *S* are based on *F*^2^, conventional *R*-factors *R* are based on *F*, with *F* set to zero for negative *F*^2^. The threshold expression of *F*^2^ > σ(*F*^2^) is used only for calculating *R*-factors(gt) *etc*. and is not relevant to the choice of reflections for refinement. *R*-factors based on *F*^2^ are statistically about twice as large as those based on *F*, and *R*- factors based on ALL data will be even larger.

### Fractional atomic coordinates and isotropic or equivalent isotropic displacement parameters (Å^2^)

**Table:**

	*x*	*y*	*z*	*U*_iso_*/*U*_eq_	
O1	−0.00370 (8)	0.34647 (13)	−0.09298 (6)	0.0694 (3)	
O2	0.04862 (6)	0.20854 (10)	0.05531 (6)	0.0513 (2)	
C1	−0.27750 (10)	0.22424 (17)	0.10333 (11)	0.0614 (3)	
H1A	−0.2984	0.1604	0.1558	0.074*	
C2	−0.35219 (11)	0.3310 (2)	0.04219 (14)	0.0770 (4)	
H2A	−0.4230	0.3383	0.0536	0.092*	
C3	−0.32221 (12)	0.4264 (2)	−0.03548 (14)	0.0793 (4)	
H3A	−0.3728	0.4978	−0.0766	0.095*	
C4	−0.21631 (11)	0.41640 (17)	−0.05268 (11)	0.0653 (3)	
H4A	−0.1961	0.4815	−0.1050	0.078*	
C5	−0.14040 (9)	0.30857 (13)	0.00867 (8)	0.0481 (3)	
C6	−0.17032 (8)	0.21122 (13)	0.08690 (8)	0.0461 (2)	
C7	−0.02835 (9)	0.29314 (14)	−0.01389 (8)	0.0495 (3)	
C8	0.02859 (8)	0.16880 (12)	0.15967 (7)	0.0439 (2)	
H8A	0.0341	0.2713	0.2019	0.053*	
C9	−0.08557 (9)	0.09595 (13)	0.14927 (8)	0.0484 (3)	
H9A	−0.0896	−0.0113	0.1138	0.058*	
H9B	−0.1002	0.0786	0.2194	0.058*	
C10	0.11974 (9)	0.05047 (14)	0.21118 (9)	0.0528 (3)	
H10A	0.1079	0.0185	0.2804	0.063*	
H10B	0.1147	−0.0498	0.1683	0.063*	
C11	0.23470 (9)	0.12051 (13)	0.22511 (9)	0.0506 (3)	
C12	0.30019 (11)	0.08497 (17)	0.15378 (11)	0.0647 (3)	
H12A	0.2725	0.0181	0.0952	0.078*	
C13	0.40664 (12)	0.1470 (2)	0.16750 (15)	0.0809 (4)	
H13A	0.4488	0.1209	0.1185	0.097*	
C14	0.44906 (13)	0.2457 (2)	0.25261 (17)	0.0870 (5)	
H14A	0.5200	0.2870	0.2622	0.104*	
C15	0.38502 (15)	0.2832 (2)	0.32398 (15)	0.0894 (5)	
H15A	0.4131	0.3509	0.3820	0.107*	
C16	0.27839 (13)	0.22086 (18)	0.31051 (11)	0.0708 (4)	
H16A	0.2365	0.2475	0.3597	0.085*	

### Atomic displacement parameters (Å^2^)

**Table:**

	*U*^11^	*U*^22^	*U*^33^	*U*^12^	*U*^13^	*U*^23^
O1	0.0738 (6)	0.0907 (7)	0.0453 (4)	−0.0159 (5)	0.0161 (4)	0.0086 (4)
O2	0.0496 (4)	0.0622 (5)	0.0443 (4)	−0.0036 (3)	0.0148 (3)	0.0004 (3)
C1	0.0497 (6)	0.0677 (7)	0.0686 (7)	−0.0063 (5)	0.0169 (5)	−0.0006 (6)
C2	0.0477 (6)	0.0826 (9)	0.0997 (11)	0.0023 (6)	0.0136 (7)	0.0030 (8)
C3	0.0588 (7)	0.0751 (9)	0.0955 (10)	0.0062 (6)	−0.0020 (7)	0.0134 (8)
C4	0.0631 (7)	0.0630 (7)	0.0640 (7)	−0.0052 (6)	0.0008 (5)	0.0107 (6)
C5	0.0504 (6)	0.0486 (5)	0.0430 (5)	−0.0087 (4)	0.0045 (4)	−0.0041 (4)
C6	0.0464 (5)	0.0478 (5)	0.0438 (5)	−0.0073 (4)	0.0086 (4)	−0.0074 (4)
C7	0.0566 (6)	0.0529 (6)	0.0387 (5)	−0.0123 (4)	0.0098 (4)	−0.0043 (4)
C8	0.0483 (5)	0.0457 (5)	0.0380 (5)	−0.0015 (4)	0.0100 (4)	−0.0036 (4)
C9	0.0506 (5)	0.0508 (5)	0.0453 (5)	−0.0044 (4)	0.0138 (4)	0.0014 (4)
C10	0.0533 (6)	0.0488 (6)	0.0564 (6)	0.0023 (4)	0.0121 (4)	0.0014 (5)
C11	0.0500 (5)	0.0460 (5)	0.0527 (6)	0.0073 (4)	0.0047 (4)	0.0031 (4)
C12	0.0598 (7)	0.0671 (7)	0.0680 (7)	0.0025 (6)	0.0152 (6)	−0.0048 (6)
C13	0.0600 (8)	0.0819 (9)	0.1042 (11)	0.0051 (7)	0.0252 (8)	0.0072 (9)
C14	0.0520 (7)	0.0795 (10)	0.1203 (14)	−0.0013 (7)	−0.0019 (8)	0.0093 (10)
C15	0.0777 (10)	0.0835 (10)	0.0908 (11)	−0.0065 (8)	−0.0181 (8)	−0.0153 (8)
C16	0.0699 (8)	0.0730 (8)	0.0633 (8)	0.0056 (6)	0.0009 (6)	−0.0116 (6)

### Geometric parameters (Å, °)

**Table:**

C1—C2	1.382 (2)	C8—H8A	0.9800
C1—C6	1.405 (2)	C9—H9A	0.9700
C1—H1A	0.9300	C9—H9B	0.9700
C2—C3	1.375 (3)	C10—C11	1.517 (2)
C2—H2A	0.9300	C10—H10A	0.9700
C3—C4	1.391 (3)	C10—H10B	0.9700
C3—H3A	0.9300	C11—C16	1.378 (2)
C4—C5	1.3968 (19)	C11—C12	1.386 (2)
C4—H4A	0.9300	C12—C13	1.396 (2)
C5—C6	1.3889 (18)	C12—H12A	0.9300
C5—C7	1.496 (2)	C13—C14	1.365 (3)
C6—C9	1.5023 (19)	C13—H13A	0.9300
C7—O1	1.2054 (17)	C14—C15	1.377 (3)
C7—O2	1.3463 (17)	C14—H14A	0.9300
C8—O2	1.4559 (19)	C15—C16	1.399 (3)
C8—C9	1.521 (2)	C15—H15A	0.9300
C8—C10	1.5214 (19)	C16—H16A	0.9300
			
C2—C1—C6	120.63 (13)	C8—C9—H9A	109.5
C2—C1—H1A	119.7	C6—C9—H9B	109.5
C6—C1—H1A	119.7	C8—C9—H9B	109.5
C3—C2—C1	120.23 (14)	H9A—C9—H9B	108.1
C3—C2—H2A	119.9	C11—C10—C8	114.97 (12)
C1—C2—H2A	119.9	C11—C10—H10A	108.5
C2—C3—C4	120.14 (13)	C8—C10—H10A	108.5
C2—C3—H3A	119.9	C11—C10—H10B	108.5
C4—C3—H3A	119.9	C8—C10—H10B	108.5
C3—C4—C5	119.97 (13)	H10A—C10—H10B	107.5
C3—C4—H4A	120.0	C16—C11—C12	117.44 (14)
C5—C4—H4A	120.0	C16—C11—C10	120.88 (11)
C6—C5—C4	120.18 (13)	C12—C11—C10	121.68 (12)
C6—C5—C7	120.30 (10)	C11—C12—C13	121.74 (15)
C4—C5—C7	119.46 (12)	C11—C12—H12A	119.1
C5—C6—C1	118.84 (11)	C13—C12—H12A	119.1
C5—C6—C9	117.73 (11)	C14—C13—C12	120.23 (15)
C1—C6—C9	123.42 (11)	C14—C13—H13A	119.9
O1—C7—O2	117.57 (12)	C12—C13—H13A	119.9
O1—C7—C5	123.76 (11)	C13—C14—C15	118.86 (16)
O2—C7—C5	118.59 (11)	C13—C14—H14A	120.6
O2—C8—C9	110.33 (8)	C15—C14—H14A	120.6
O2—C8—C10	106.16 (9)	C14—C15—C16	120.99 (16)
C9—C8—C10	113.54 (11)	C14—C15—H15A	119.5
O2—C8—H8A	108.9	C16—C15—H15A	119.5
C9—C8—H8A	108.9	C11—C16—C15	120.73 (15)
C10—C8—H8A	108.9	C11—C16—H16A	119.6
C6—C9—C8	110.56 (11)	C15—C16—H16A	119.6
C6—C9—H9A	109.5	C7—O2—C8	118.82 (10)

### Hydrogen-bond geometry (Å, °)

**Table:**

*D*—H···*A*	*D*—H	H···*A*	*D*···*A*	*D*—H···*A*
C9—H9B···O1^i^	0.97	2.53	3.2922 (18)	135

Symmetry codes: (i) *x*, −*y*+1/2, *z*+1/2.
